# Supplementary material for: Single-Molecule Super-Resolution Microscopy Reveals Heteromeric Complexes of MET and EGFR upon Ligand Activation
Source: Int J Mol Sci. 2020 Apr 17;21(8):2803. doi: 10.3390/ijms21082803 (PMC7215329; doi:10.3390/ijms21082803)
Supplement: Supplementary file 1 [file ijms-21-02803-s001.pdf]

Supplementary Materials

for

Single-Molecule Super-Resolution Microscopy Reveals Heteromeric Complexes of MET and EGFR upon Ligand Activation

Marie-Lena I.E. Harwardt[1], Mark S. Schröder[1], Yunqing Li[1], Sebastian Malkusch[1], Petra Freund[1], Shashi Gupta[2], Nebojsa Janjic[2], Sebastian Strauss[3,4], Ralf Jungmann[3,4], Marina S. Dietz[1,*] & Mike Heilemann[1,*]

1 Single Molecule Biophysics, Institute of Physical and Theoretical Chemistry, Goethe University Frankfurt, Max-von-Laue-Str. 7, 60438 Frankfurt, Germany
2 SomaLogic, Inc., Boulder, CO 80301, USA
3 Department of Physics and Center for Nanoscience, Ludwig Maximilian University, 80539 Munich, Germany
4 Max Planck Institute of Biochemistry, 82152 Planegg, Germany
* Correspondence: dietz@chemie.uni-frankfurt.de (M.S.D.); heileman@chemie.uni-frankfurt.de (M.H.)

Table S1: Receptor densities of MET and EGFR on the plasma membrane of HeLa and BT-20 cells. MET and EGFR densities were determined from super-resolved DNA-PAINT images in resting, HGF, or EGF stimulated HeLa and BT-20 cells (N=6-7 cells/condition from at least three independent experiments). Errors represent standard deviations.

	**HeLa**		**BT-20**	
	**MET/µm²**	**EGFR/µm²**	**MET/µm²**	**EGFR/µm²**
unstimulated	14.1 ± 0.5	6.3 ± 1.5	11.8 ± 1.5	20.8 ± 1.6
HGF	6.3 ± 0.6	8.4 ± 1.9	8.4 ± 2.0	19.5 ± 2.7
EGF	12.4 ± 0.5	1.5 ± 0.7	9.8 ± 1.8	7.9 ± 2.7

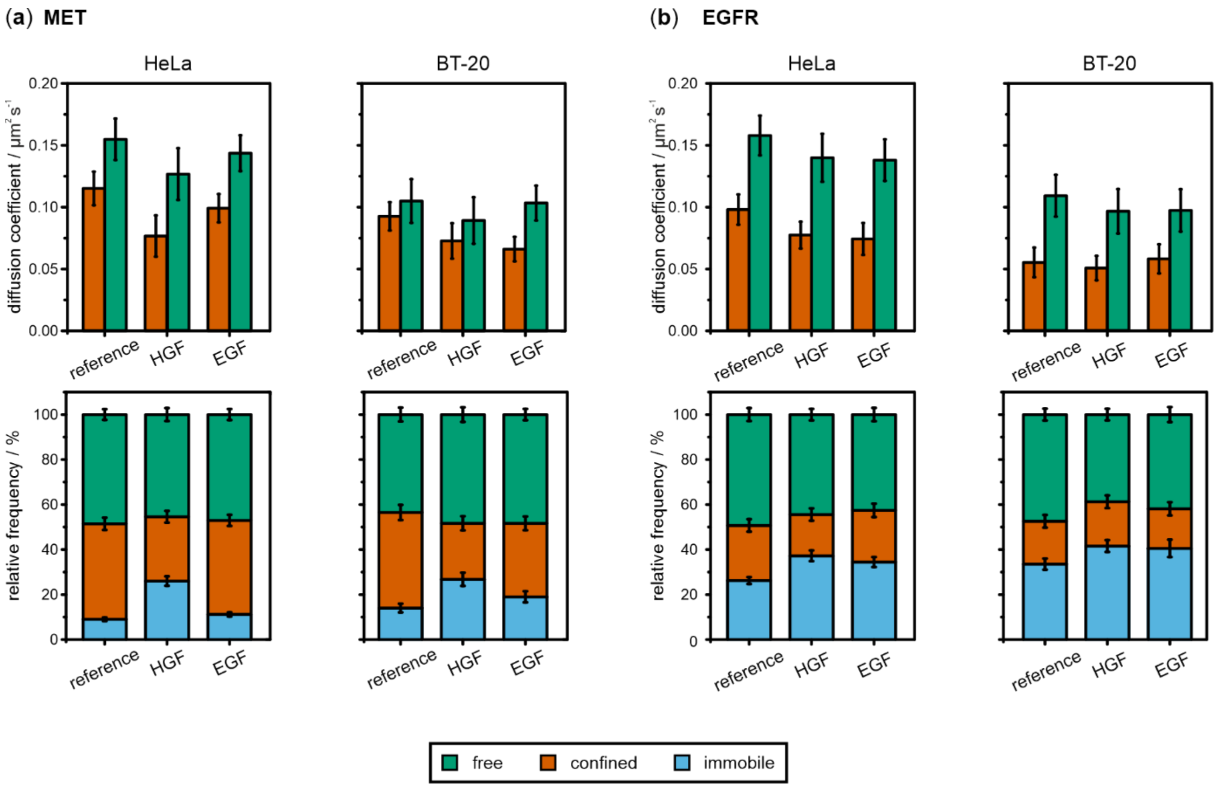

Figure S1: Diffusion coefficients and relative occurrence according to diffusion types of MET and EGFR in HeLa and BT-20 cells determined from SPT data. SPT trajectories of (a) MET and (b) EGFR in HeLa and BT-20 cells were classified either as immobile, confined, or freely diffusing according to the analysis method published byRossier et al. (2012). Mean diffusion coefficients of each population were determined and depicted as grouped stacks (upper panels in (a) and (b)) for untreated cells as well as for samples stimulated with ligands. For the same conditions, the relative frequency of each diffusion type was calculated and represented in stacked columns (lower panels in (a) and (b)). Error bars represent SEMs. N = 50, from at least three independent experiments.

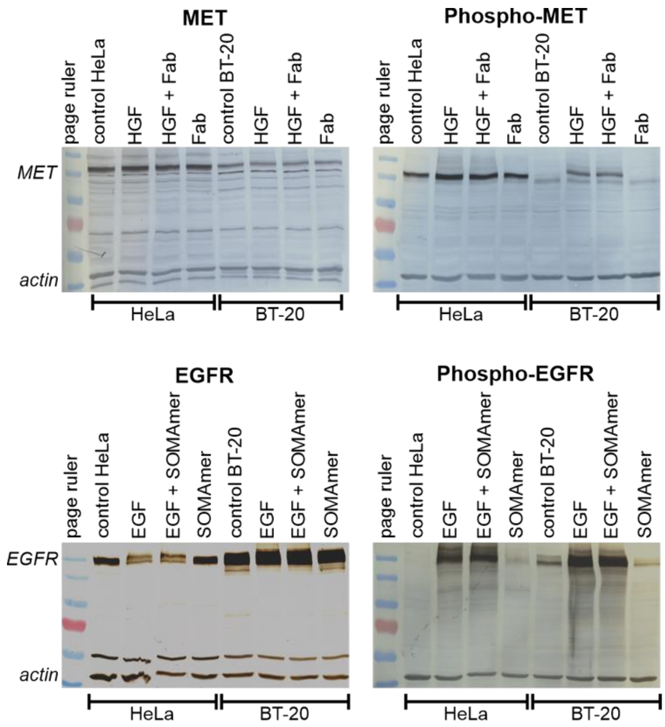

Figure S2: Western blot analysis of ligand-induced activation of MET and EGFR in HeLa and BT-20 cells after incubation with the Fab fragment or SOMAmer reagent. In order to determine the influence of the Fab antibody fragment or the SOMAmer reagent on the activation of MET or EGFR, respectively, in the absence or presence of activating ligands, western blots in HeLa and BT-20 cells were performed. Each receptor was either treated with its activating ligand, or the labeling ligand, or with a combination of both for 5 min. Antibodies against the unphosphorylated MET or EGFR were applied as a control. Antibodies against Phospho-MET or Phospho-EGFR were used to study the activation of receptors after addition of the different ligands. Actin was labeled as a housekeeping gene. Page ruler was applied as a size marker on the gel. Bands were visualized upon application of diaminobenzidine tablets with metal enhancer.

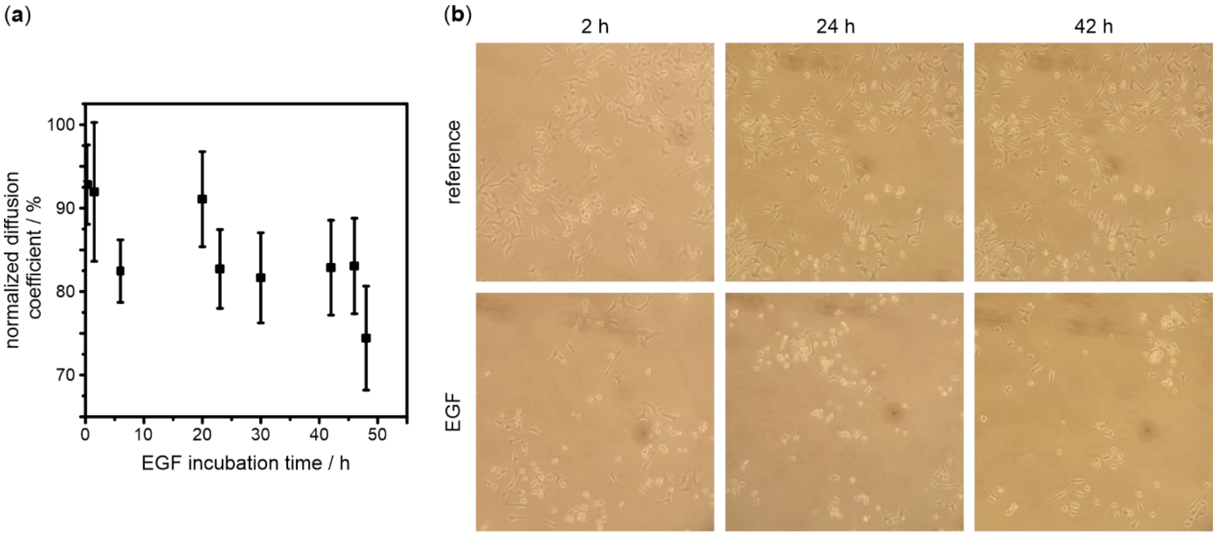

Figure S3: Effects of long-term incubation with EGF on MET diffusion dynamics and HeLa cell morphology. (a) Diffusion of MET in HeLa cells was studied by SPT after exposure to EGF for 15 min up to 48 h. Diffusion coefficients were normalized against reference samples which were not treated with any ligand and plotted against EGF incubation times. Error bars represent SEM (N = 20, from at least three independent experiments). (b) The morphology of HeLa cells exposed to EGF was monitored for up to 42 h. Images of untreated and treated cells are shown for incubation times of 2, 24, and 42 h.

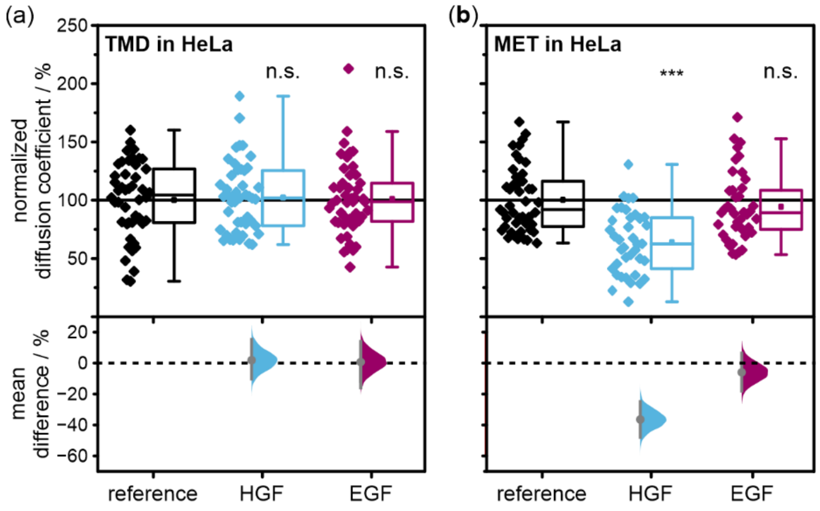

Figure S4: Diffusion dynamics of TMD in transfected HeLa cells measured by SPT do not change upon stimulation with HGF and EGF. HeLa cells were transfected with a plasmid encoding for mEGFP-TMD. (a) mEGFP-TMD was tracked using a FluoTag®-Q nanobody labeled with Abberior Star 635P which binds the extracellular mEGFP. (b) MET was tracked using Fab-ATTO 647N. Untreated cells served as reference. Stimulation of MET or EGFR was achieved by treating cells either with HGF (light blue) or EGF (purple), respectively. Diffusion coefficients of mEGFP-TMD and MET were determined in resting and ligand-stimulated cells (N = 46-47 for mEGFP-TMD, N = 38-46 for MET, each from at least three independent experiments). All diffusion coefficients were normalized against the respective untreated reference. The box plots of normalized diffusion coefficients display the 5th percentile, 25th percentile, median (line), mean (square), 75th percentile, and 95th percentile. Results of two-sample t-tests for comparison of ligand-treated cells with the reference are depicted above the box plots ($p > 0.05$ no significant difference between populations (n.s.), $p < 0.001$ highly significant difference (***)). The lower y axes of the graphs depict the distribution of mean differences (Ho et al. 2019) of each condition in comparison to the unstimulated sample. The mean difference is represented as a grey dot; each 95% confidence interval of the mean difference distribution is indicated by vertical grey error bars.

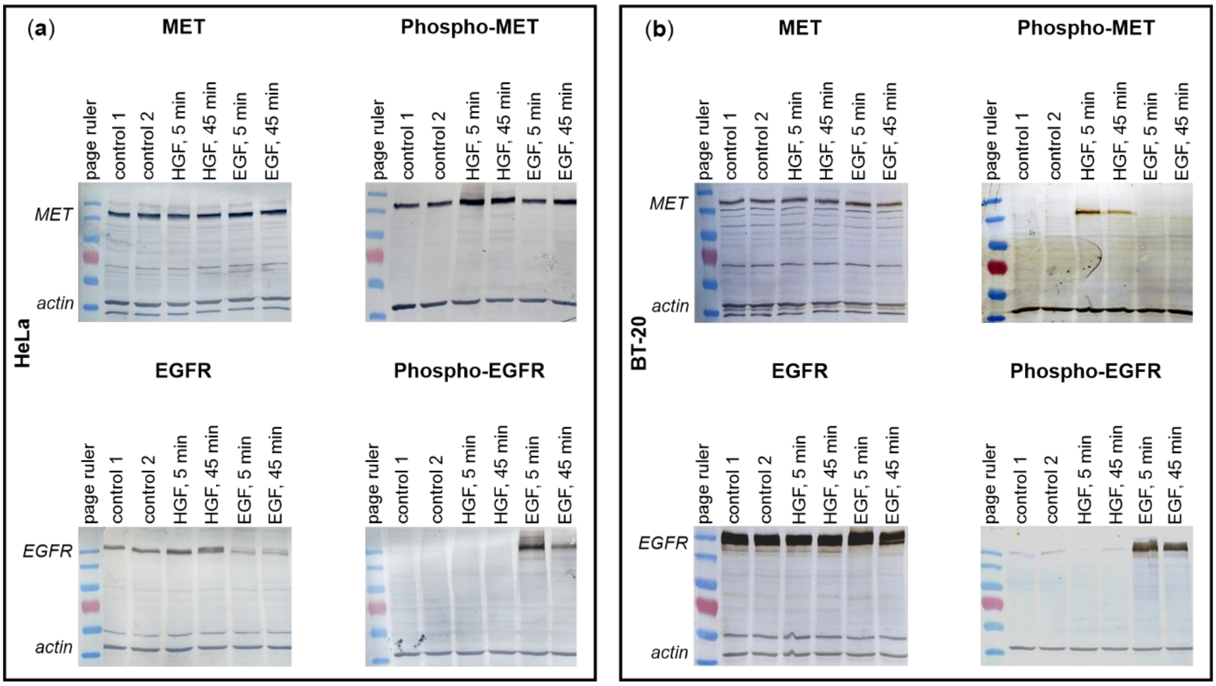

Figure S5: Western blot analysis of MET and EGFR in HeLa and BT-20 cells after ligand stimulation. (a) HeLa or (b) BT-20 cells were either left untreated or stimulated with HGF or EGF for 10 min. Western blots of these samples were developed either applying antibodies against unphosphorylated MET and EGFR as a control or the phosphorylated receptors for examination of activation. Actin was labeled as housekeeping gene. Page ruler was applied as a size marker on the gel. Bands were visualized by chemiluminescence detection (illumination time = 2 s).
